# Correlation between chest computed tomography findings and pulmonary function test results in the post-recovery phase of COVID-19

**DOI:** 10.31744/einstein_journal/2023AO0288

**Published:** 2023-12-06

**Authors:** Gustavo Borges da Silva Teles, Eduardo Kaiser Uruhary Nunes Fonseca, Julia Capobianco, Patricia Yokoo, Marcela Emer Egypto Rosa, Telma Antunes, Carolina Silva Bernardes, Tatiane Cristina Marques, Rodrigo Caruso Chate, Gilberto Szarf

**Affiliations:** 1 Hospital Israelita Albert Einstein São Paulo SP Brazil Hospital Israelita Albert Einstein , São Paulo , SP , Brazil .; 2 Grupo Fleury São Paulo SP Brazil Grupo Fleury , São Paulo , SP , Brazil .

**Keywords:** COVID-19, Coronavirus infections, SARS-CoV-2, Respiratory function tests, Pulmonar fibrosis, Pulmonary diffusing capacity, Carbon monoxide, Tomography, X-ray computed

## Abstract

**Objective:**

The radiological and functional lung sequelae in COVID-19 survivors remain unclear. We compared the chest computed tomography findings of COVID-19 patients with normal and abnormal pulmonary function test results in the post-recovery phase.

**Methods:**

The data of consecutive patients who underwent pulmonary function tests and chest computed tomography within 14 days after recovery from COVID-19 at two medical centers between May and October 2020 were collected retrospectively. Two thoracic radiologists who were blinded to the clinical information and pulmonary function test results classified the patients according to the computed tomography features, evidence of fibrotic-like changes, and semi-quantitative quantification of the extent of pulmonary abnormalities. The clinical characteristics and computed tomography findings of patients with normal pulmonary function test results were compared with those of patients with abnormal results.

**Results:**

A total of 101 COVID-19 survivors, comprising 48 ambulatory and 53 hospitalized patients, were included at a median of 95 days from initial symptom onset. Computed tomography revealed fibrotic-like changes in 10.9% of patients. A reduction in the diffusion capacity of carbon monoxide was the most common lung function abnormality (19.8%). Abnormal diffusion capacity of carbon monoxide was associated with the presence and extension of lung opacities on chest computed tomography scans and fibrotic pulmonary abnormalities. The sensitivity, specificity, and accuracy of reduced diffusion capacity of carbon monoxide for detecting fibrotic-like pulmonary changes on chest computed tomography scans were 72.7%, 87.8%, and 86.1%, respectively.

**Conclusion:**

Our study suggests that the presence of an abnormal diffusion capacity of carbon monoxide in the post-recovery phase of COVID-19 is associated with a greater risk of long-term parenchymal lung disease, as evidenced by the presence of fibrotic-like changes on chest computed tomography scans, such as traction bronchiectasis and architectural distortion.

## Summary

In Brief

Teles et al. evaluated the chest computed tomography findings of COVID-19 survivors with normal and abnormal pulmonary function test results in the post-recovery phase. They demonstrated that an abnormal carbon monoxide diffusing capacity is associated with the extension of lung opacities on chest computed tomography and the presence of fibrotic-like changes, such as traction bronchiectasis and architectural distortion.

## Abstract (key points)

Highlights

Computed tomography revealed fibrotic-like lung changes in 10.9% of patients.

Higher computed tomography scores were associated with a reduced DLCO (median computed tomography score, 6 versus 3; p=0.016).

Fibrotic-like computed tomography changes were more prevalent in patients with DLCO impairment (p<0.001).

Reduced DLCO had sensitivity and specificity of 73% and 88%, respectively, for predicting fibrotic-like computed tomography changes.

## Graphical abstract

**Figure:**
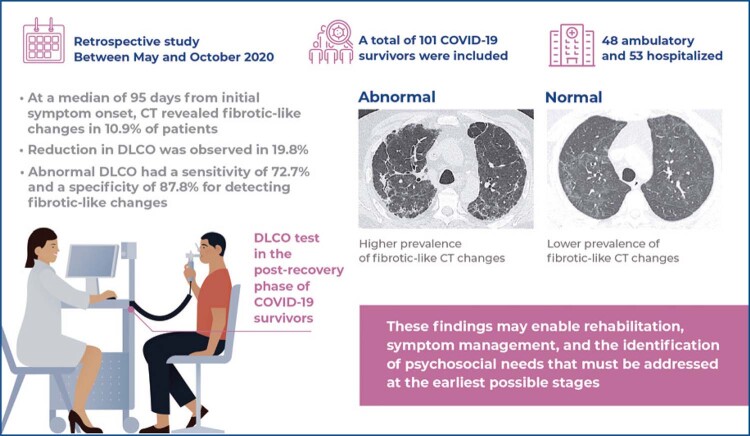

## INTRODUCTION

Coronavirus disease (COVID-19) has been associated with multiple organ damage. Pneumonia is the most common manifestation of COVID-19 infection, with the intensity ranging from mildly asymptomatic cases to cases with critical respiratory failure requiring ventilatory support. ^( 1 )^ Preliminary data have revealed that more than half of the adult survivors of SARS-CoV-2 infection have post-acute COVID-19, also known as “long COVID.” ^( 2 , 3 )^ Respiratory symptoms, such as cough and dyspnea, are reported in nearly 30% of patients with post-acute COVID-19, including those with mild infections that do not necessitate hospitalization. ^( 4 , 5 )^

Survivors of COVID-19 may have pulmonary function abnormalities that persist for weeks to months after the resolution of the acute illness. ^( 6 )^ In addition, parenchymal abnormalities are frequently detected during lung imaging after infection, particularly in severe cases. ^( 7 )^ These findings are consistent with those of previous studies on the long-term respiratory sequelae of acute respiratory failure and observations from previous severe coronavirus outbreaks. ^( 8 , 9 )^

The radiological and functional lung sequelae of COVID-19 can affect the survivors’ quality of life and remain unclear. Reports on the association between lung sequelae detected on chest computed tomography (CT) and pulmonary function test (PFT) results in patients who have recovered from COVID-19 have increased recently; nevertheless, many uncertainties remain.

## OBJECTIVE

We compared the chest computed tomography findings of patients with COVID-19 who had normal and abnormal pulmonary function test results in the post-recovery phase.

## METHODS

This retrospective study was conducted at two medical centers in São Paulo, Brazil, and was approved by the local Institutional Review Board. Written informed consent was obtained from all patients. The data of consecutive patients who underwent PFT, spirometry, and diffusion capacity of carbon monoxide (DLCO) assessment after recovery from COVID-19 between May and October 2020 were collected retrospectively. Patients with no chest CT findings within 14 days of the PFT or incomplete PFT findings were excluded. Confirmed COVID-19 was defined as a positive reverse transcriptase polymerase chain reaction test result from a nasopharyngeal or oropharyngeal swab.

The following clinical information was extracted from the electronic medical records of the patients: age, sex, body mass index, smoking status, presence of comorbidities (systemic arterial hypertension, diabetes, chronic obstructive pulmonary disease [COPD], asthma, or another pulmonary disease), persistent symptoms (dyspnea, cough, fever, and anosmia), and requirement for hospitalization and intensive care unit (ICU).

### Chest CT scans

Multidetector CT scanners with 64, 80, or 320 detector rows (Brilliance, Philips Medical Systems, Eindhoven, Netherlands; Somaton Definition, Siemens Healthcare, Erlangen, Germany; Aquilion Prime and Aquilion ONE, Canon Medical Systems, Tochigi, Japan) were used to acquire the chest CT images. All CT images were obtained in the supine position during end inspiration without intravenous contrast material. The acquisition parameters were as follows: reconstructed slice thickness, 1mm; voltage of 80–120 kVp; and automatic milliampere settings (range–10–440 mA).

### Analysis of the CT images

Two thoracic radiologists (with 13 and 4 years of experience in interpreting chest images, respectively), who were blinded to the clinical information and PFT results, reviewed the chest CT images independently in a standard clinical Picture Archiving and Diagnostic System workstation. The CT features of each patient were assessed and classified as follows according to the Fleischner Society Glossary: ^( 10 )^ ground-glass opacities (GGO), consolidation, reticulation, interlobular septal thickening, parenchymal bands, architectural distortion, traction bronchiectasis, honeycombing, pleural effusion, and lymphadenopathy. Fibrotic-like changes were defined as the presence of traction bronchiectasis, architectural distortion, or honeycombing. ^( 11 , 12 )^ The final classification was determined based on a consensus between the readers.

A semi-quantitative CT score was used to quantify the extent of pulmonary abnormalities, as described previously. ^( 13 )^ Briefly, each lung was divided into the upper (above the carina), middle, and lower (below the inferior pulmonary vein) zones. Each zone was evaluated to determine the percentage of lung involvement on a scale of 0 to 4, with each score indicating the following: 0, 0% involvement; 1, <25% involvement; 2, 25% to less than 50% involvement; 3, 50% to less than 75% involvement, and 4, ≥75% involvement. The overall CT score was the summation of the scores of all six lung zones. The maximum possible score is 24.

### Pulmonary function tests

Pulmonary function tests were performed by professionally trained respiratory technicians using the Vyntus One Pulmonary Function System (Vyaire Medical, USA) and interpreted by an experienced pulmonologist according to the current recommendations. ^( 14 )^ On account of the COVID-19 restrictions, PFTs were limited to spirometry and diffusing capacity of the lungs for carbon monoxide (DLCO) assessment. ^( 15 )^ Spirometric parameters included forced vital capacity (FVC), forced expiratory volume in the first second (FEV1), FEV1/FVC ratio, and the transfer factor for carbon monoxide (TLCO). The PFT parameters were expressed as a percentage of the predicted value (%) and considered impaired if the values were below the lower limit of normal, according to the Global Lung Function Initiative 2012 reference equations for spirometry ^( 16 )^ and the Global Lung Function Initiative 2017 reference equations for DLCO. ^( 17 )^

### Statistics

Continuous variables are presented as mean ± standard deviation or median and interquartile range (IQR), whereas categorical variables are presented as counts and percentages. The clinical characteristics and CT features of the patients with normal and abnormal PFT results were compared using the Student’s *t* -test and Mann-Whitney´s test (continuous variables) or χ ^2^ test and Fisher’s exact test (binary/categorical variables). A p value of <0.05 indicated statistical significance. The sensitivity, specificity, positive predictive value (PPV), negative predictive value (NPV), and accuracy were calculated, considering impaired DLCO as a positive predictor for fibrotic-like CT changes. Data were analyzed using SPSS version 21.0 (SPSS Inc. Chicago, IL).

## RESULTS

**Figure 1:**
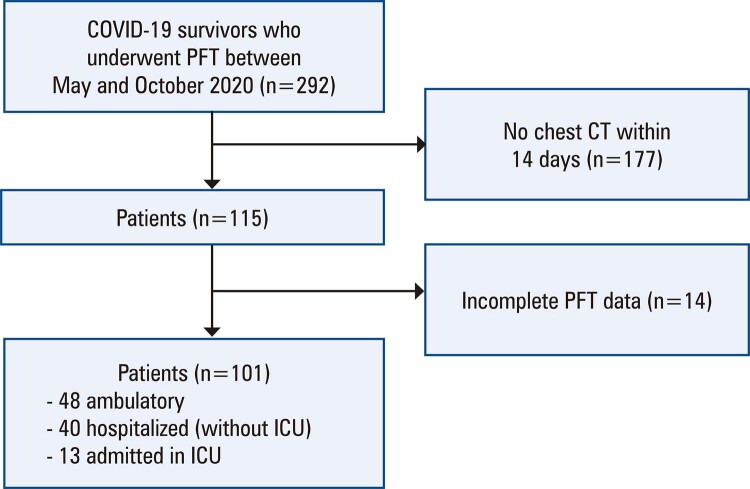
Illustration of study flow

PFT: pulmonary function tests; CT: computed tomography; ICU: intensive care unit.

This study included 101 COVID-19 survivors (48 ambulatory and 53 hospitalized, among whom 13 required ICU admission) at a median of 95 days (range: 71–123; IQR: 85–110) from initial symptom onset ( Figure 1 ). Persistence of symptoms was observed in 32.7% of patients. Dyspnea and cough were the most frequently observed symptoms. Most patients had abnormal chest CT findings (70.3%), and the most common tomographic findings were ground-glass opacities (63.3%), reticular opacities (44.6%), and parenchymal bands (30.7%). Fibrotic-like CT changes were observed in 11 patients (10.9%). However, honeycombing was not observed. A reduction in DLCO% predicted was the most common abnormality in lung function (19.8% of patients), followed by abnormal FEV1% and FVC% predicted (13.9% and 12.9%, respectively) ( Table 1 ).

**Table 1:** Clinical and tomographic characteristics stratified by normal or abnormal pulmonary function tests results

Variable	DLCO% predicted			FVC% predicted			FEV1% predicted			FEV1/FVC% predicted			Total

	Normal	Abnormal	p value	Normal	Abnormal	p value	Normal	Abnormal	p value	Normal	Abnormal	p value	
Mean age - years (SD)	50.4 (13.9)	62.3 (9.9)	<0.001	51.5 (14.1)	61.4 (9.4)	0.144	52.2 (14.4)	56.3 (10.0)	0.155	52.1 (13.7)	61.6 (13.9)	0.042	52.7 (14.0)
Sex (%)			0.019*			0.268*			>0.999 ^#^				
Male	54 (88.5)	7 (11.5)		54 (88.5)	7 (11.5)		52 (85.2)	9 (14.8)		56 (91.8)	5 (8.2)	0.700 ^#^	61
Female	28 (19.6)	12 (80.4)		34 (85.0)	6 (15.0)		35 (87.5)	5 (12.5)		38 (95.0)	2 (5.0)		40
SAH (%)			0.322 ^#^			0.241 ^#^			0.269 ^#^			0.605 ^#^	
Yes	13 (72.2)	5 (17.8)		14 (77.8)	4 (22.2)		14 (77.8)	4 (22.2)		16 (88.9)	2 (11.1)		18
No	69 (83.1)	14 (16.9)		74 (89.2)	9 (10.8)		73 (88.0)	10 (12.0)		78 (94.0)	5 (6.0)		83
Diabetes (%)			0.683 ^#^			>0.999 ^#^			>0.999 ^#^			>0.999 ^#^	
Yes	9 (90.0)	1 (10.0)		9 (90.0)	1 (10.0)		9 (90.0)	1 (10.0)		10 (100.0)	0 (0.0)		10
No	73 (80.2)	18 (19.8)		79 (86.8)	12 (13.2)		78 (85.7)	13 (14.3)		84 (92.3)	7 (7.7)		91
Obesity - BMI >30 (%)			0.388 ^#^			0.176 ^#^			0.342 ^#^			>0.999 ^#^	
Yes	24 (88.9)	3 (11.1)		26 (96.3)	1 (3.7)		25 (92.6)	2 (7.4)		25 (92.6)	2 (7.4)		27
No	58 (78.4)	16 (21.6)		62 (83.8)	12 (16.2)		62 (83.8)	12 (16.2)		69 (93.2)	5 (6.8)		74
Smoking history (%)			0.001 ^#^			0.089 ^#^			0.108 ^#^			0.118 ^#^	
Never/former	79 (85.9)	13 (14.1)		82 (89.1)	10 (10.9)		81 (88.0)	11 (12.0)		87 (94.6)	5 (5.4)		92
Current	3 (33.3)	6 (66.7)		6 (66.7)	3 (33.3)		6 (66.7)	3 (33.3)		7 (77.8)	2 (22.2)		9
COPD (%)			0.342 ^#^			>0.999 ^#^			>0.999 ^#^			>0.999 ^#^	
Yes	1 (50.0)	1 (50.0)		2 (100.0)	0 (0.0)		2 (100.0)	0 (0.0)		2 (100.0)	0 (0.0)		2
No	81 (81.8)	18 (18.2)		86 (86.9)	13 (13.1)		85 (85.9)	14 (14.1)		92 (92.9)	7 (7.1)		99
Asthma (%)			0.999 ^#^			0.651 ^#^			0.011 ^#^			0.003 ^#^	
Yes	10 (83.3)	2 (16.7)		10 (83.3)	2 (16.7)		7 (58.3)	5 (41.7)		8 (66.7)	4 (33.3)		12
No	72 (80.9)	17 (19.1)		78 (87.6)	11 (12.4)		80 (89.9)	9 (10.1)		86 (96.6)	3 (3.4)		89
Hospitalization (%)			0.045 ^#^			0.195 ^#^			0.842*			0.706 ^#^	
Yes	39 (73.6)	14 (16.4)		44 (83.0)	9 (17.0)		46 (86.8)	7 (13.2)		50 (94.3)	3 (5.7)		53
No	43 (89.6)	5 (10.4)		44 (91.7)	4 (8.3)		41 (85.4)	7 (14.6)		44 (91.7)	4 (8.3)		48
ICU (%)			0.023*			0.341 ^#^			0.686 ^#^			0.589 ^#^	
Yes	9 (60.0)	6 (40.0)		13 (86.7)	2 (13.3)		14 (93.3)	1 (6.7)		15 (100.0)	0 (0.0)		15
No	73 (84.9)	13 (15.1)		75 (87.2)	11 (12.8)		73 (84.9)	13 (15.1)		79 (91.9)	7 (8.1)		86
Persistent symptoms (%)			0.286 ^#^			>0.999 ^#^			0.768 ^#^			>0.999 ^#^	
Yes	29 (87.9)	4 (12.1)		29 (87.9)	4 (12.1)		28 (84.8)	5 (15.2)		31 (93.9)	2 (6.1)		33
No	53 (77.9)	15 (22.1)		59 (86.8)	9 (13.2)		59 (86.8)	9 (13.2)		63 (92.6)	5 (7.4)		68
CT appearance													
Any pulmonary opacity			0.011 ^#^			0.750 ^#^			>0.999 ^#^			0.671 ^#^	
Yes	53 (74.6)	18 (25.4)		61 (85.9)	10 (14.1)		61 (85.9)	10 (14.1)		65 (91.5)	6 (8.5)		71
No	29 (96.7)	1 (3.3)		27 (90.0)	3 (10.0)		26 (86.7)	4 (13.3)		29 (96.7)	1 (3.3)		30
Median CT score (IQR)	3 (6)	6 (4)	0.005 ^&^	4 (6)	6 (9)	0.113 ^&^	4 (6)	6 (8)	0.291 ^&^	5.5 (8)	6 (4)	0.448 ^&^	6 (8)
Fibrotic-like changes			<0.001 ^#^			0.149 ^#^			0.648 ^#^			0.529 ^#^	
Yes	3 (27.3)	8 (72.7)		8 (72.7)	3 (27.3)		9 (81.8)	2 (18.2)		10 (90.9)	1 (9.1)		11
No	79 (87.8)	11 (12.2)		80 (88.9)	10 (11.1)		78 (86.7)	12 (13.3)		84 (93.3)	6 (6.7)		90
Total	82	19		88	13		87	14		94	7		101

Student *t* -test; * χ ^2^ test; ^#^ Fisher’s exact test; ^&^ Mann-Whitney´s test.

SAH: systemic arterial hypertension; ICU: intensive care unit; BMI: body mass index; COPD: chronic obstructive pulmonary disease; IQR: interquartile range; DLCO: diffusion capacity of carbon monoxide; FVC: forced vital capacity; FEV1: forced expiratory volume in 1 second; CT: computed tomography.

### Correlation between clinical parameters and PFT results

Abnormal DLCO% predicted was more prevalent among older patients (mean age, 62.3 *versus* 50.4 years; p<0.001), females (p=0.019), current smokers (p=0.001), and patients who were hospitalized (p=0.045) or in the ICU (p=0.023). Asthma was associated with a reduction in FEV1% (p=0.011) and FEV1/FVC% predicted (p=0.003). No statistically significant differences were observed between the persistence of symptoms and altered PFT results.

The CT scores of patients who were hospitalized or in the ICU were higher than those of ambulatory patients (median CT score, 6 *versus* 2.5; p=0.037). Fibrotic-like CT changes were observed significantly more frequently in patients who were in the ICU than in ambulatory patients or those who were hospitalized (p=0.005).

### Correlation between CT features and PFT results

Reduced DLCO% predicted was the only pulmonary function parameter associated with abnormal chest CT findings (p=0.011), CT score (median CT score, 6 *versus* 3; p=0.016), and the presence of fibrotic-like CT changes (p<0.001).

**Figure 2:**
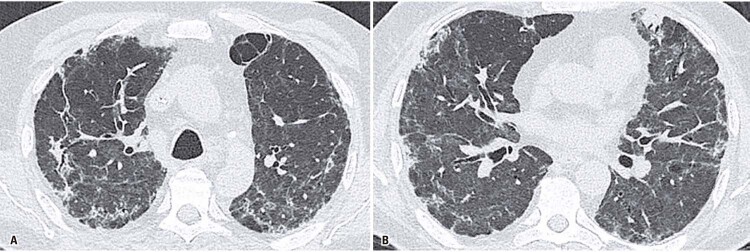
A and B) Fibrotic-like changes in a 62 years-old male patient, 92 days after COVID-19 initial symptoms onset, with abnormal diffusion capacity of carbon monoxide (53% of the predicted value). Chest computed tomography shows ground-glass opacities, linear and reticular abnormalities, discrete traction bronchiectasis and slight parenchymal architectural distortion

**Figure 3:**
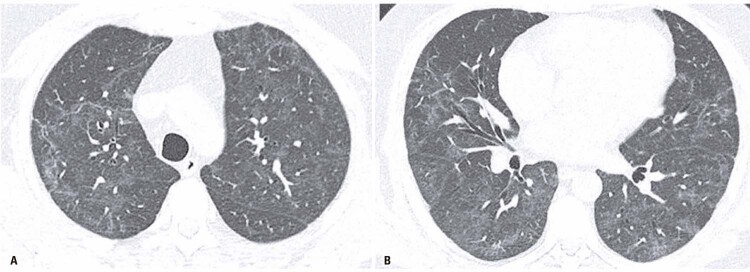
A and B) Non-fibrotic changes in a 49 years-old male patient, 85 days after symptoms onset, with normal diffusion capacity of carbon monoxide (106% of the predicted value). Chest computed tomography shows ground glass opacities with minimal linear abnormalities. There is no evidence of traction bronchiectasis, architectural distortion or honeycombing

Among abnormal chest CT findings, the presence of reticular opacities (p=0.003), bronchial dilatation (p=0.005), and architectural distortion (p<0.001) were also significantly more prevalent in patients with impaired DLCO (Figures 2 and 3 ). No statistically significant differences were observed between the distribution of lung opacities on chest CT and the presence of ground-glass opacities, consolidation, or parenchymal bands in these patients ( Table 2 ).

**Table 2:** Pulmonary computed tomography features stratified by normal or abnormal diffusion capacity of carbon monoxide % predicted

Variable	DLCO% predicted			p value

	Normal	Abnormal	Total	
Ground glass opacities				0.566
Yes	50 (74.6)	17 (25.4)	67	
No	4 (100.0)	0 (0.0)	4	
Consolidation				0.999
Yes	6 (75.0)	2 (25.0)	8	
No	48 (76.2)	15 (23.8)	63	
Reticular opacities				0.003
Yes	30 (65.2)	16 (34.8)	46	
No	25 (96.2)	1 (3.8)	26	
Parenchymal bands				0.148*
Yes	21 (67.7)	10 (32.3)	31	
No	33 (82.5)	7 (17.5)	40	
Bronchial dilatation				0.005
Yes	3 (33.3)	6 (66.7)	9	
No	51 (82.3)	11 (17.7)	62	
Architectural distortion				<0.001
Yes	1 (12.5)	7 (87.5)	8	
No	53 (84.1)	10 (15.9)	63	
Distribution				0.866*
Peripheral	33 (76.7)	10 (23.3)	43	
Non-peripheral	21 (75.0)	7 (25.0)	28	
Total	54	17	71	

Fisher’s exact test; * χ ^2^ test.

DLCO: diffusion capacity of carbon monoxide.

The sensitivity, specificity, PPV, NPV, and accuracy of reduction in DLCO% predicted as a positive test for predicting fibrotic-like CT changes were 72.7% (95%CI: 39.0–94.0%), 87.8% (95%CI: 79.2–93.7%), 42.1% (95%CI: 27.3–58.5%), 96.3% (95%CI: 90.9–98.6%), and 86.1% (95%CI: 77.8–92.2%), respectively ( Table 3 ).

**Table 3:** Performance results of abnormal diffusion capacity of carbon monoxide for fibrotic computed tomography changes

Variable	Test performance				

	Sensitivity (95%CI)	Specificity (95%CI)	PPV (95%CI)	NPV (95%CI)	Accuracy (95%CI)
Abnormal DLCO	72.7	87.8	42.1	96.3	86.1
	(39.0-94.0)	(79.2-93.7)	(27.3-58.5)	(90.9-98.6)	(77.8-92.2)

PPV: positive predictive value; NPV: negative predictive value; 95%CI: confidence interval; DLCO: diffusion capacity of carbon monoxide.

## DISCUSSION

Our study demonstrates that an abnormal DLCO% predicted in COVID-19 survivors at a median of 95 days from the initial symptom onset was associated with the presence and extension of lung opacities on chest CT scans and the presence of fibrotic pulmonary abnormalities. We also demonstrated that reduced DLCO had a sensitivity, specificity, and accuracy of 72.7%, 87.8%, and 86.1%, respectively, for detecting the presence of fibrotic-like pulmonary changes on chest CT scans.

Impaired DLCO was also found to be the most common functional lung abnormality in COVID-19 survivors at the 30-day, 3-month, 6-month, and 1-year follow-up visits in previous studies. ^( 18 - 22 )^ Zhao et al. ^( 19 )^ evaluated chest CT and PFT results in 55 patients with mild or moderate COVID-19 3 months after the infection. Similar to our results, DLCO impairment and abnormal CT scores were observed in nine (16.3%) and 39 (70.9%) of the 55 patients, respectively. Pure GGO and interstitial thickening were the most common CT features. A reduction in the PFT values was more prevalent among patients with abnormal CT findings (30.8%) than among those with normal CT findings (12.5%); however, the difference was not statistically significant.

Balbi et al. ^( 20 )^ evaluated the post-discharge CT findings and PFT results of 91 patients with severe COVID-19 at a median of 105 days from symptom onset. However, the prevalence of abnormal chest CT findings (81.3%) and fibrotic lung abnormalities (72.5%) was higher than that in our study (70.3% and 10.9%, respectively), possibly due to the higher severity of the disease in their sample. In their analysis, the DLCO and FVC percentages were significantly lower in patients with CT abnormalities, and the percentage of lung involvement on CT images was found to have a significant negative correlation with the DLCO percentage.

The pulmonary sequelae at the 6-month follow-up of COVID-19 survivors were assessed by Han et al., ^( 21 )^ who found a higher prevalence of pulmonary fibrotic-like CT changes (35% of the participants) than in our study; however, their study evaluated patients with more severe disease. Abnormal DLCO was observed in 26% of the patients, which occurred more frequently in participants with pulmonary fibrotic-like changes than in those without fibrotic-like changes, similar to our results.

Although the optimal timing for follow-up imaging to assess radiological clearance in patients with COVID-19 remains unknown, a recognized clinical guideline ^( 23 )^ considers a 12-week time point to be optimal as it provides sufficient time for imaging resolution while also ensuring that non-resolving changes are addressed sufficiently early. This follow-up algorithm recommends clinical assessment followed by chest radiography and PFT 12 weeks after the onset of COVID-19 pneumonia. Patients with abnormal chest radiographs and/or physiological impairments detected by PFT should undergo high-resolution chest CT.

We included COVID-19 survivors at a median time interval of 95 days. Our results reinforce the hypothesis that functional assessment may be useful for determining which patients should be further investigated for potentially life-limiting complications of COVID-19, such as pulmonary fibrosis. We found that DLCO impairment was associated with higher CT scores and the presence of fibrotic-like pulmonary changes on chest CT.

Our study has some limitations. First, this was a retrospective analysis and the indications for PFT and chest CT after COVID-19 were based on clinical judgment, as no published guidelines were available at the time of the study. Furthermore, our sample included a small number of patients with severe COVID-19, resulting in fewer abnormal PFT results and fibrotic-like changes on chest CT scans. Lastly, chest CT did not include expiratory images, which limited the assessment of air trapping and small airway involvement, consequences that have recently been described in COVID-19 survivors. ^( 24 )^

## CONCLUSION

In summary, our study builds on previous evidence that the presence of an abnormal diffusion capacity of carbon monoxide in the post-recovery phase of COVID-19 is associated with a greater risk of long-term parenchymal lung disease, which is characterized by the presence of fibrotic-like changes on chest computed tomography scans, such as traction bronchiectasis and architectural distortion. These findings may enable rehabilitation, symptom management, and the identification of psychosocial needs that must be addressed at the earliest possible stages.
